# Heterogeneous Sensory Innervation and Extensive Intrabulbar Connections of Olfactory Necklace Glomeruli

**DOI:** 10.1371/journal.pone.0004657

**Published:** 2009-02-27

**Authors:** Renee E. Cockerham, Adam C. Puche, Steven D. Munger

**Affiliations:** Department of Anatomy and Neurobiology, University of Maryland School of Medicine, Baltimore, Maryland, United States of America; Tokyo Medical and Dental University, Japan

## Abstract

The mammalian nose employs several olfactory subsystems to recognize and transduce diverse chemosensory stimuli. These subsystems differ in their anatomical position within the nasal cavity, their targets in the olfactory forebrain, and the transduction mechanisms they employ. Here we report that they can also differ in the strategies they use for stimulus coding. Necklace glomeruli are the sole main olfactory bulb (MOB) targets of an olfactory sensory neuron (OSN) subpopulation distinguished by its expression of the receptor guanylyl cyclase GC-D and the phosphodiesterase PDE2, and by its chemosensitivity to the natriuretic peptides uroguanylin and guanylin and the gas CO_2_. In stark contrast to the homogeneous sensory innervation of canonical MOB glomeruli from OSNs expressing the same odorant receptor (OR), we find that each necklace glomerulus of the mouse receives heterogeneous innervation from at least two distinct sensory neuron populations: one expressing GC-D and PDE2, the other expressing olfactory marker protein. In the main olfactory system it is thought that odor identity is encoded by a combinatorial strategy and represented in the MOB by a pattern of glomerular activation. This combinatorial coding scheme requires functionally homogeneous sensory inputs to individual glomeruli by OSNs expressing the same OR and displaying uniform stimulus selectivity; thus, activity in each glomerulus reflects the stimulation of a single OSN type. The heterogeneous sensory innervation of individual necklace glomeruli by multiple, functionally distinct, OSN subtypes precludes a similar combinatorial coding strategy in this olfactory subsystem.

## Introduction

**Figure 1:**
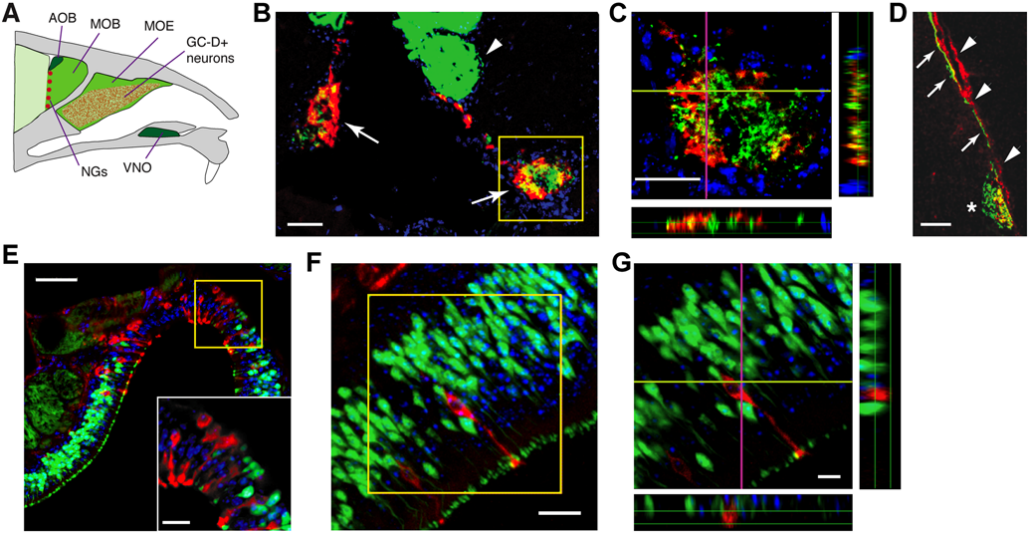
Nonoverlapping expression of PDE2 and OMP in necklace glomeruli and MOE. (A) The mouse main and accessory olfactory systems. The main system (bright green) includes the main olfactory epithelium (MOE) and main olfactory bulb (MOB). GC-D+ neurons (red stippling) are dispersed amongst canonical OSNs in restricted regions of the MOE. Necklace glomeruli (NGs; red circles) are in the caudal MOB. The accessory system (dark green) is comprised of the vomeronasal organ (VNO) and accessory olfactory bulb (AOB). Adapted from [3], with permission from Elsevier. (B) NGs in paired MOBs of an *OMP-tauEGFP* ^+/−^ *::Gucy2d-Mapt-lacZ* ^+/−^ mouse. EGFP, green; PDE2, red. Arrowhead: canonical MOB glomerulus; arrows: necklace glomeruli; box, shown in (C). (C) Orthogonal views (right, bottom; vertical, horizontal lines are planes of optical section) of a single NG confocal z-stack showing little overlap of EGFP-positive (green) and PDE2-positive (red) axons. (D) MOB section from *Gucy2d-Mapt-lacZ* ^+/−^ mouse, immunostained for OMP (green), β-gal (red). Arrows: OMP-positive axons; arrowheads: β-gal-positive axons. (E) MOE dorsal recess of *OMP-tau-EGFP* ^+/−^ mouse shows clusters of PDE2-positive/EGFP-negative (red) and PDE2-negative/GFP-positive (green) neurons. Yellow box: area magnified 2-fold, inset (F) Single PDE2-positive OSN (red) in the MOE of an *OMP-tau-EGFP* ^+/−^ mouse. EGFP, green; box, shown in (G). (G) Orthogonal views in MOE of single PDE-positive/EGFP-negative (red) neuron and multiple PDE2-negative/EGFP-positive (green) neurons. Confocal z-stack (B,C,E–G); single optical section (D). Scale bars: 50 µm (B–D); 10 µm (E–G). Blue, DAPI.

The main olfactory bulbs (MOBs) of rodents each have 9–15 “necklace” glomeruli, which encircle the posterior bulb like beads on a chain [1]–[6] (**Figure 1A**). The necklace glomeruli receive functional input from a small population of sensory neurons in the nose that express the receptor guanylyl cyclase GC-D, the cyclic nucleotide-gated channel subunit A3 and phosphodiesterase 2 (PDE2) [1]–[4], [7]–[11]. Unlike canonical OSNs, which use a cAMP-mediated signaling cascade to transduce odors, GC-D-positive neurons lack many components of the cAMP-based system [8], [9] and instead employ a cGMP-mediated mechanism [10]. We recently reported that mouse olfactory sensory neurons (OSNs) that express GC-D show excitatory responses to urine, a rich source of semiochemicals for many mammals, and to two novel chemostimuli, the natriuretic peptide hormones uroguanylin and guanylin [10]. Others report that these cells are involved in CO_2_ sensing as well [11]. A mixture of uroguanylin and guanylin activates all GC-D-positive neurons, but these peptides never activate other MOE neurons [10]. Moreover, deletion of the gene encoding GC-D (*Gucy2d*) abolished all MOE responses to these peptides [10]. Necklace glomeruli receive afferent neural signals reflecting stimulation of the main olfactory epithelium (MOE) by uroguanylin, guanylin and CO_2_ [10], [11]. They also respond to the putative pheromones 2-heptanone and 2′5′-dimethylpyrazine [12], even though these two molecules do not stimulate GC-D-positive OSNs [10]. The neural circuitry and coding strategies used to process these diverse chemosignals within the MOB are unknown.

In canonical MOB glomeruli, odors evoke distinct patterns of activity [13], [14]. Each odor molecule can activate multiple odor receptors (ORs), and OSNs expressing the same OR converge onto just a few glomeruli [15]–[20]. Canonical OSNs seem to each express a single type of OR protein (e.g., [21]–[24], though see [25]); thus, any individual glomerulus receives functionally homogeneous OSN input [13]–[15], [26]. As a result, it is believed that the pattern of glomerular activation establishes a combinatorial code representing the identity of individual odors [15]. However, it was unknown whether this coding strategy is conserved throughout the main olfactory system, including in the GC-D-positive neurons and necklace glomeruli.

## Results

Functional studies of GC-D-positive neurons [10], [11] or the necklace glomeruli [10]–[12] support roles in the detection of peptidergic, volatile and/or gaseous stimuli. Given the molecular complexity of the chemostimuli detected by GC-D-positive OSNs, we wondered if the necklace glomeruli, in contrast to canonical MOB glomeruli, might integrate more than one type of olfactory input. Therefore, we characterized the afferent innervation of the necklace glomeruli. PDE2 is a robust and specific marker for GC-D-positive neurons and their axons [8], [10], [11], so we used PDE2 fluorescent immunohistochemistry (IHC) to visualize necklace glomeruli in mice expressing enhanced green fluorescent protein (EGFP) under the control of the olfactory marker protein (OMP) promoter (*Omp-tau-EGFP* mice) (**Figure 1**). Olfactory marker protein (OMP), a molecule heavily expressed in mature canonical OSNs [27], is also expressed in OSN axons innervating the necklace glomeruli [28]. We confirmed that the necklace glomeruli are innervated by OMP-positive neurons (**Figure 1B and C**). However, confocal reconstruction of single necklace glomeruli indicated that axons of EGFP-positive (i.e., OMP-expressing) neurons were distinct from those of PDE2-positive (i.e., GC-D-expressing) neurons (**Figure 1B and C**). Double labeling with PDE2 and OMP antisera gave similar results (Supplemental data, **Figure S1A**). Axons of OMP-expressing and GC-D-expressing OSNs form separate fascicles before innervating the necklace glomeruli (**Figure 1D**). These surprising results indicate that, in distinct contrast to the canonical MOB glomeruli, each necklace glomerulus is innervated by two distinct sensory neuron populations: one expressing PDE2 and GC-D, and one expressing OMP.

Resolution of individual axons is difficult in glomeruli. To confirm that GC-D-positive/PDE2-positive OSNs do not express OMP, we performed PDE2 IHC in the main olfactory epithelium (MOE) of *Omp-tau-EGFP* mice. Expression of PDE2 and EGFP is mutually exclusive: we saw no neurons that coexpressed these markers (**Figure 1E, F and G**). Previous studies reported that PDE2-positive OSNs could be found both clustered and as solitary neurons in the MOE [8], though this study did not examine the expression of OMP in these cells. We found PDE2-positive OSNs in clusters largely devoid of EGFP-positive OSNs (**Figure 1E**), or as solitary cells within regions rich in EGFP-positive OSNs (**Figure 1F**). Orthogonal views of confocal z-stacks confirmed that PDE2-positive cells do not express EGFP (**Figure 1G**). Similar results were seen with OMP/PDE2 double labeling (Supplementary data, **Figure S1B and C**). Taken together, the glomerular and MOE labeling demonstrates that OMP-positive/PDE2-negative axons that innervate the necklace glomeruli must arise from neurons other than GC-D-positive/PDE2-positive OSNs.

**Figure 2:**
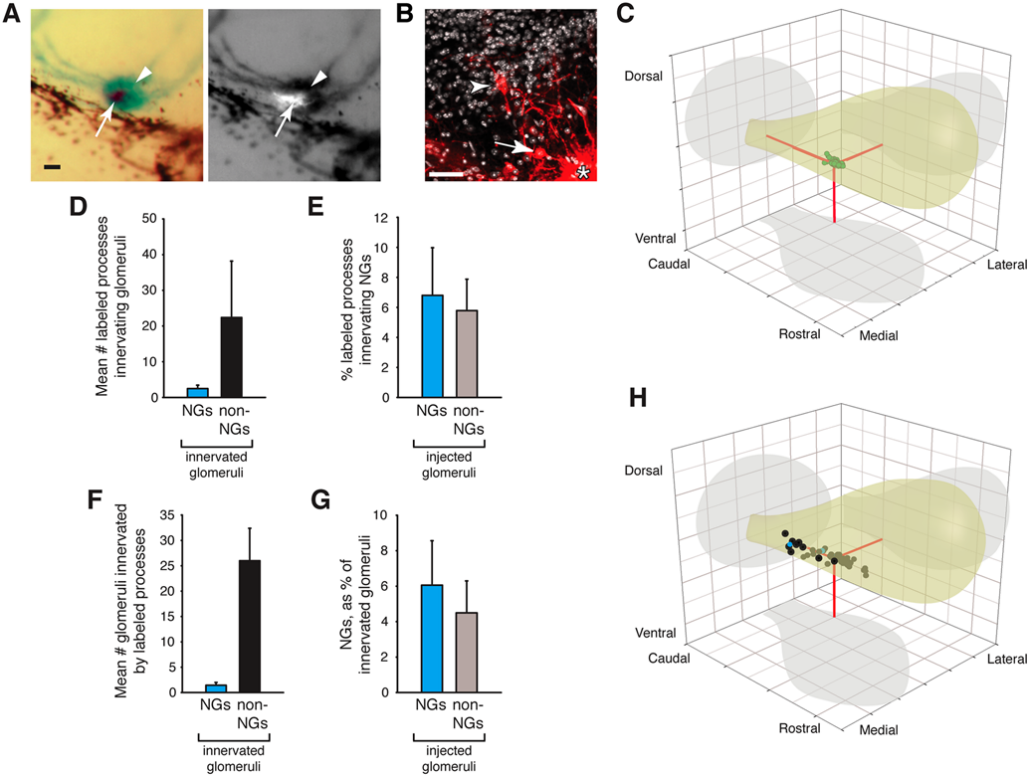
Intrabulbar connections with necklace glomeruli. (A) Brightfield (left) and fluorescent (right) whole mount images after X-gal histochemistry of a necklace glomerulus (NG; arrowhead) injected with DiI (arrow). Scale bar, 25 µm. (B) DiI labeled cells associated with a DiI-injected NG (asterisk). Arrow, periglomerular cell; arrowhead, mitral cell. Scale bar, 25 µm. (C) Distribution of labeled cell bodies (green) from a representative NG injection. Red lines indicate position of the injection point within the cell cluster and on the shadows (gray) of the generic MOB schematic (yellow). Each grid square = 500 µm^2^. (D) Labeled cell processes innervating glomeruli (NGs, blue; non-NGs, black) after DiI injections of single NGs (n = 7). (E) Mean percentage of labeled processes innervating NGs after DiI injection of NGs (blue) or non-NGs (gray). No significant differences between groups by Student's t test. (F) NGs and non-NGs innervated by labeled processes after DiI injections of single NGs (n = 7). (G) Necklace glomeruli as a percentage (mean) of total glomeruli innervated by labeled processes after DiI injection of NGs (blue) or non-NGs (gray). No significant differences between groups by Student's t test. (H) Distribution of innervated glomeruli (NGs, blue; non-NGs, black) from same NG injection as in (C). All error bars, s.e.m.

The different patterns of sensory innervation observed for canonical MOB glomeruli [26] and the necklace glomeruli, and the consequences of these differences for olfactory coding, beg the question of whether the necklace glomeruli are part of, or are separate from, the main olfactory system. For example, no direct connections have been observed between MOB glomeruli and glomeruli of the accessory olfactory bulb (AOB) [29], suggesting that chemosensory information handled by MOB and AOB glomeruli are processed independently. In contrast, extensive interglomerular connections within the MOB through juxtaglomerular interneurons contribute to the processing of odor information [14]. To investigate the extent of interglomerular connections between necklace glomeruli and other glomeruli we performed iontophoretic injections (5–10 µm in diameter) [30] of individual ventral necklace glomeruli (n = 7) with the lipophilic dye DiI in *Gucy2d-Mapt-lacZ* ^+/−^ (n = 5) and *Gucy2d-Mapt-lacZ* ^−/−^ (n = 2) mice (**Figure 2A**). *Gucy2d-Mapt-lacZ* mice express a tau-β-galactosidase (β-gal) fusion protein under the control of the *Gucy2d* promoter [10], enabling precise targeting of individual necklace glomeruli after X-gal histochemistry. No differences in necklace glomeruli position [10], number [10] or interglomerular connections (data not shown) were observed between *Gucy2d-Mapt-lacZ* ^+/−^ and ^−/−^ mice. Only bulbs in which the dye injection was restricted to the targeted glomerulus (**Figure 2A**; Supplemental data, **Figure S2**) were analyzed.

After a period of 10–20 days to allow for dye transport, we sectioned the bulbs and visualized labeled MOB neurons by confocal microscopy (**Fig. 2B**). The positions of labeled cell bodies were mapped in three dimensions relative to the injected glomerulus [30]. Labeled mitral cells, which project to higher brain centers, exhibited typical MOB morphology: they displayed extensive lateral dendrites and a single apical dendrite (data not shown). Cell bodies of juxtaglomerular interneurons (e.g., periglomerular cells, short axon cells) were found as far as 1377 µm from the injection site (median distance = 193.8 µm; mean distance = 214.4 µm). A representative distribution of labeled cell bodies from one necklace glomerulus injection is shown (**Figure 2C**).

We also mapped all labeled cell processes that innervated glomeruli. Surprisingly, only 5.8% of these processes (2.1±0.9 processes per injection) innervated other β-gal-positive glomeruli (i.e., other necklace glomeruli) (**Figure 2D and E**). The remaining processes (35.0±15.8 processes per injection) innervated β-gal-negative (i.e., non-necklace) glomeruli (**Figure 2D**). DiI injections of β-gal-negative glomeruli (n = 4) in the posterior MOB gave similar results (**Figure 2E**; Supplemental data, **Figure 3**). Only 5.2% of glomeruli innervated by processes labeled by a necklace glomerulus injection (1.4±0.57 innervated glomeruli per injection) were β-gal-positive (**Figure 2F and G**). Again, injections of β-gal-negative glomeruli (n = 4) gave similar results (**Figure 2G**; Supplemental data, **Figure S3**). A representative distribution of innervated glomeruli from one necklace glomerulus injection is shown (**Figure 2H**). The numerous connections between necklace and non-necklace glomeruli within the MOB indicate that afferent signals (e.g., activation of GC-D-expressing neurons) received by necklace glomeruli may be processed in the context of other olfactory information.

**Figure 3:**
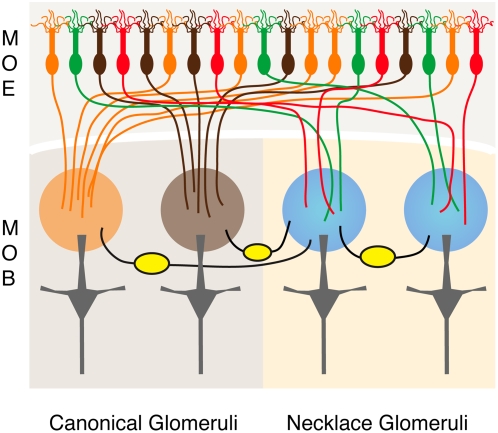
Schematic of sensory neuron and interneuron innervation of necklace glomeruli. The MOE contains numerous sensory neurons (top), including: canonical OSNs that all express OMP but which express different ORs (orange, brown); GC-D/PDE2-expressing OSNs (red); and OMP-positive/PDE4A-negative neurons (green). Canonical OSNs expressing the same OR converge on the individual canonical glomeruli (orange, brown circles) in the MOB, providing homogeneous sensory input to those structures. GC-D/PDE2-positive neurons innervate necklace glomeruli (blue circles), as do OMP-positive/PDE4A-negative neurons. Juxtaglomerular interneurons in the glomerular layer (yellow ovals) connect individual necklace glomeruli with other necklace glomeruli and with near and distant canonical glomeruli. Mitral cells (gray, bottom) innervating necklace glomeruli display a typical MOB mitral cell morphology.

## Discussion

The extensive and unexpected connections between necklace and non-necklace glomeruli provide an opportunity for olfactory information integrated within the necklace glomeruli to be processed in the context of other odor signals (**Figure 3**). The relatively anterior position of many of the MOB glomeruli that share interneuron connections with the necklace glomeruli (e.g., **Figure 2H**) strongly suggests that necklace glomeruli are heavily connected to canonical MOB glomeruli (i.e., those innervated by OSNs expressing ORs and components of an olfactory cAMP cascade [31]). If so, information about the detection of semiochemicals (e.g., pheromones or social cues) might be integrated with more general odor information, though the impact of these interglomerular connections on the physiological output of mitral and/or tufted cells (the second order projection neurons of the olfactory system) remains to be determined. However, this extensive connectivity with other glomeruli of the MOB clearly positions the necklace glomeruli as novel components of the MOB, not as anatomically and functionally distinct CNS structures analogous to the AOB. Even so, connections between PDE2-positive necklace glomeruli and other glomeruli of the posterior MOB could serve a more specialized role, perhaps related to the detection of semiochemicals. Glomeruli in this region appear to form several whole or partial “necklaces” with either distinct or overlapping sensory inputs (e.g., [32]). The molecular and functional characterization of those glomeruli that share interneurons with the necklace glomeruli should help illuminate the context in which olfactory information is processed by this subsystem.

Since the GC-D-positive/PDE2-positive neurons and the OMP-positive neurons innervating the necklace glomeruli are distinct populations, we conclude that individual necklace glomeruli receive information about more than one type of chemosensory stimulus through the convergence of OSNs expressing different chemosensory receptor types (GC-D and carbonic anhydrase II are putative chemosensory receptors in the GC-D-positive neurons [10], [11], [33], while the receptor(s) in the OMP-positive neurons are unknown). This differs dramatically from canonical MOB glomeruli, which are targeted by OSNs expressing the same OR and thus receive homogeneous odor input [15], [17], [26], [34], [35] (**Fig. 3**). This homogeneous input is a central component of the combinatorial strategy thought to underlie odor coding in the main olfactory system [15]. Of course, it remains possible that canonical glomeruli also receive inputs from more than one OSN subpopulation. However, ultrastructural analyses of canonical glomeruli suggest this is unlikely [26].

The population of necklace glomeruli may be functionally heterogeneous. GC-D-positive neurons can be grouped into three functionally distinct subpopulations: while some are responsive to both guanylin and uroguanylin, others respond to only one or the other of these peptides [10]. It is unclear whether these functionally distinct sensory cell types target different subsets of necklace glomeruli. However, the sensory innervation of necklace glomeruli may be even more complex. For example, neurons of the Grueneberg ganglion, a small group of chemosensory neurons at the rostral tip of the nasal cavity that mediate alarm pheromone detection [36] and are sensitive to cool temperatures [37], project to the posterior MOB but innervate only ∼5 dorsomedial glomeruli [38]–[42]. Many Grueneberg ganglion neurons express both PDE2 and OMP [43]. Since all PDE2-positive glomeruli are innervated by both GC-D-positive/PDE2-positive/OMP-negative OSNs [10] and by OMP-positive/PDE2-negative OSNs (**Fig. 1**), these Grueneberg ganglion axons likely represent a third afferent input to a subset of necklace glomeruli. The chemosensory heterogeneity of GC-D-positive neurons and other neurons innervating subsets of necklace glomeruli suggests an additional complexity to olfactory processing by the necklace glomeruli.

Combinatorial odor coding by the canonical OSNs and MOB glomeruli may underlie the olfactory system's ability to recognize an immense array of possible olfactory cues. In contrast, the olfactory subsystem that includes GC-D-positive/PDE2-positive OSNs and the necklace glomeruli responds with high sensitivity to just a few stimuli [10]–[12]. As chemostimuli that activate GC-D-positive OSNs do not activate other OSNs [10], [11], OMP-positive neurons innervating individual necklace glomeruli must respond to stimuli other than guanylin [10], uroguanylin [10] or CO_2_ [11]. The ligands for the OMP-positive neurons that innervate the necklace glomeruli (which lack a key marker for canonical OSNs, PDE4A [8], [44]) are not known. However, the volatile pheromones 2′,5′-dimethylpyrazine and 2-heptanone – which, like uroguanylin, are found in urine – are intriguing candidates. These compounds upregulate the expression of immediate early genes in cells surrounding necklace glomeruli in the absence of canonical cAMP-mediated signaling [12], but do not activate GC-D-positive neurons [10]. Our observation that necklace glomeruli receive heterogeneous afferent input suggests a resolution to this apparent conflict. The innervation of individual necklace glomeruli by OSNs that respond to different olfactory stimuli suggests that each necklace glomerulus may operate as a coincidence detector, perhaps for semiochemicals. Further anatomical and functional studies are needed to determine whether individual projection neurons (i.e., mitral and/or tufted cells) associated with these necklace glomeruli integrate stimuli from both groups of sensory neurons or are compartmentalized to receive only a single type of input. In any case, the combination of discrete chemosensory stimuli [10]–[12], novel sensory transduction mechanisms [2], [10], [11] and targeting by distinct chemoreceptor inputs shown here indicate that the necklace glomeruli are part of a specialized olfactory subsystem with a different strategy for olfactory coding.

## Materials and Methods

### Immunohistochemistry

Immunohistochemistry (IHC) for OMP [27], PDE2 [8] and β-galactosidase (β-gal) was performed in *Omp-tau-EGFP^+/−^::Gucy2d-Mapt-lacZ* ^+/−^ ([10], [45]; n = 4), C57BL/6J (WT; n = 3) or *Gucy2d-Mapt-lacZ* ^+/−^ (n = 1; *Gucy2d* encodes GC-D) adult mice, as described previously [10], [46]. Briefly, after fixation with 4% paraformaldehyde by intracardial perfusion, paired MOBs were sectioned (horizontal plane) by cryostat at 16 µm thickness; MOE was sectioned (coronal plane) by cryostat at 10 or 16 µm thickness. Sections were mounted on glass slides for IHC processing. Primary antisera were used at 1∶1000 (rabbit-anti-OMP; gift of F. Margolis [47]) or 1∶500 (goat-anti-PDE2; Santa Cruz Biotechnology [10], [12]) and visualized with Cy2-conjugated, donkey-anti-rabbit or Cy3-conjugated, donkey-anti-goat secondary antibodies (both Jackson Immunoresearch), respectively. Enhanced green fluorescent protein (EGFP) fluorescence was visualized directly. Individual confocal z-stack optical sections were taken at 1 µm spacing using an Olympus Fluoview 500 confocal microscope. We examined at least 18 necklace glomeruli per animal (n = 6 mice). For MOE sections, we examined at least four MOE sections containing PDE2-positive OSNs per animal (n = 7 mice): two sections containing clusters of PDE2-positive OSNs within dorsocaudal recesses; and two sections from more rostral regions of the MOE, which contain relatively few PDE2-positive OSNs [7], [8], [10], [11], [28]. Confocal images, including orthogonal views, were processed using Olympus Fluoview v5.0 software.

### Glomerular injections, DiI labeling and mapping of labeled cells

Glomerular injections were performed as described [30]. Briefly, *Gucy2d-Mapt-lacZ* ^+/−^ (n = 5) and *Gucy2d-Mapt-lacZ* ^−/−^ (n = 2) adult mice were intracardially perfused with cold phosphate buffered saline, followed by brief perfusion with 1% paraformaldehyde in 0.1 M phosphate buffer (PFA; pH 7.4). Next, chromogenic detection of β-galactosidase (β-gal) activity was performed by perfusing with 10 ml of β-gal chromogenic solution (200 µM K_3_Fe(CN)_6_, 200 µM K_4_Fe(CN)_6_, and 1 mg/mL 5-bromo-4-chloro-3-indolyl-beta-D-galactopyranoside (X-Gal). Brains were removed and a single MOB glomerulus per bulb injected iontophoretically with DiI (1 mg/ml in ethanol, 0.5–2.0 µA, tip diameter 1.5 µm). Brains were incubated at 30°C in PFA for 10–20 days, serially sectioned (40 µm in the coronal plane) and incubated in DAPI before coverslipping. Sections were visualized at high magnification and the position of labeled cell bodies or processes plotted on a low magnification DAPI image. The rostrocaudal distance of each cell body or process was measured from the section number, and the dorsoventral and mediolateral coordinates measured using Corel graphics programs.

All animal experiments were approved by the University of Maryland School of Medicine IACUC.

## Supporting Information

Figure S1 Immunolocalization of OMP and PDE2 in necklace glomeruli and MOE. (A) Orthogonal view of a single necklace glomerulus in *Gucy2d-Mapt-lacZ+/−* mouse, immunostained for OMP (green), PDE2 (red). (B) Immunostaining of WT MOE shows PDE2-positive/OMP-negative (red) and OMP-positive/PDE2-negative (green) OSNs. Box: shown in (C). (C) Orthogonal views in MOE of single PDE2-positive/OMP-negative neuron and multiple OMP-positive/PDE2-negative neurons. Scale bars: 50 um (A); 25 um (B,C). All are confocal z-stacks. Blue, DAPI.
(2.90 MB TIF)

Figure S2 Restriction of DiI injections to single necklace glomeruli. (A) Brightfield and (B) fluorescent images of the same MOB section showing a single necklace glomerulus stained by X-gal histochemistry (blue) and injected with DiI (red). White, DAPI (pseudocolored). Arrow, tract of iontophoresis pipet. Scale bar, 50 um.
(1.01 MB TIF)

Figure S3 Cell labeling through DiI injections of non-necklace glomeruli. (A) Brightfield (left) and fluorescent (right) whole mount images after X-gal histochemistry of a non-necklace glomerulus in the posterior MOB injected with DiI (arrow). Arrowhead, nearby necklace glomerulus. Scale bar, 25 um. (B) Labeled cell processes innervating glomeruli (NGs, blue; non-NGs, black) after DiI injections of single beta-galactosidase-negative glomeruli (n = 4). Of these processes, 3.4% innervated NGs (2.0±1.0 processes per injection). Error bars, s.e.m. (C) NGs and non-NGs innervated by labeled processes after DiI injections of single beta-galactosidase-negative glomeruli (n = 4). Of innervated glomeruli, 2.5% were NGs (1.0±0.6 per injection). Error bars, s.e.m.
(2.35 MB DOC)
