# Supplementary figures and images for: Heterogeneous Sensory Innervation and Extensive Intrabulbar Connections of Olfactory Necklace Glomeruli
Source: PLoS One. 2009 Feb 27;4(2):e4657. doi: 10.1371/journal.pone.0004657 (PMC2645502; doi:10.1371/journal.pone.0004657)

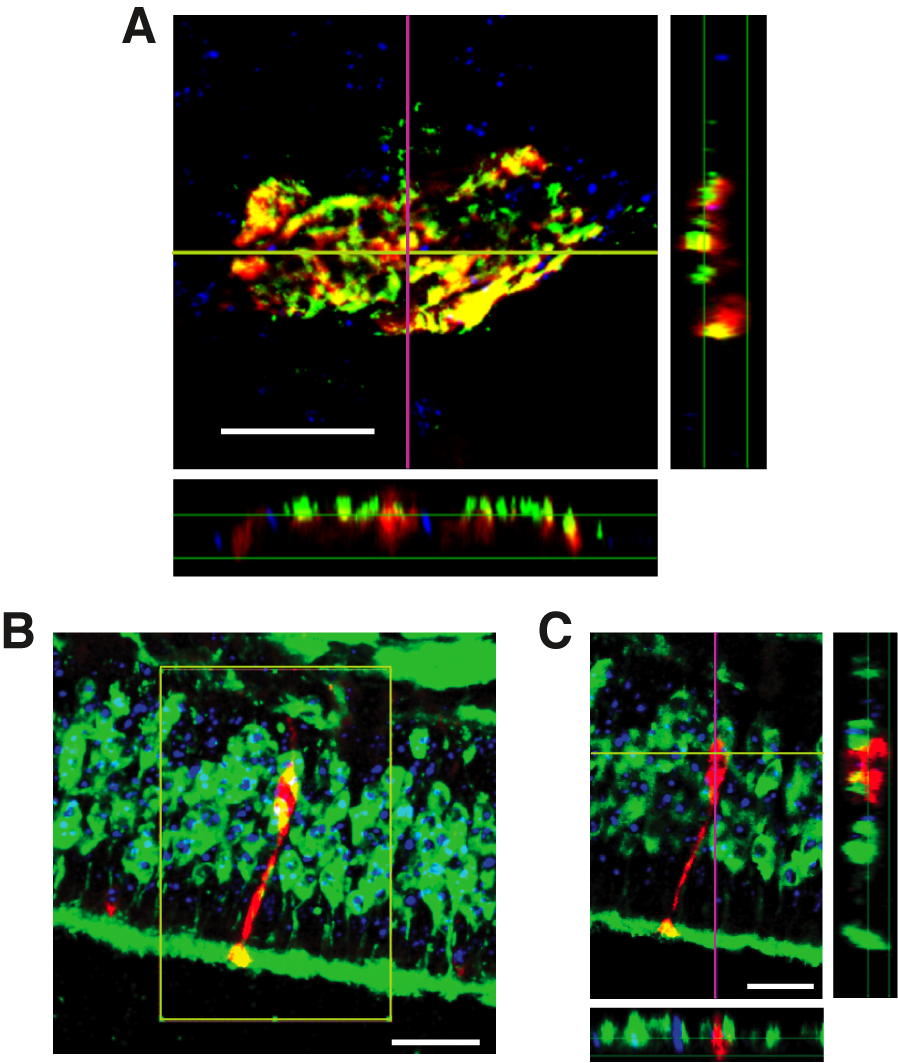

Supplement: Figure S1 — Immunolocalization of OMP and PDE2 in necklace glomeruli and MOE. (A) Orthogonal view of a single necklace glomerulus in Gucy2d-Mapt-lacZ+/− mouse, immunostained for OMP (green), PDE2 (red). (B) Immunostaining of WT MOE shows PDE2-positive/OMP-negative (red) and OMP-positive/PDE2-negative (green) OSNs. Box: shown in (C). (C) Orthogonal views in MOE of single PDE2-positive/OMP-negative neuron and multiple OMP-positive/PDE2-negative neurons. Scale bars: 50 um (A); 25 um (B,C). All are confocal z-stacks. Blue, DAPI. (2.90 MB TIF) [file pone.0004657.s001.tif]

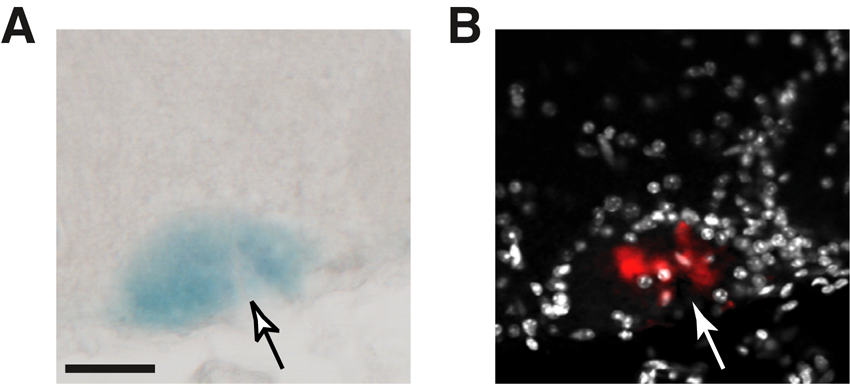

Supplement: Figure S2 — Restriction of DiI injections to single necklace glomeruli. (A) Brightfield and (B) fluorescent images of the same MOB section showing a single necklace glomerulus stained by X-gal histochemistry (blue) and injected with DiI (red). White, DAPI (pseudocolored). Arrow, tract of iontophoresis pipet. Scale bar, 50 um. (1.01 MB TIF) [file pone.0004657.s002.tif]

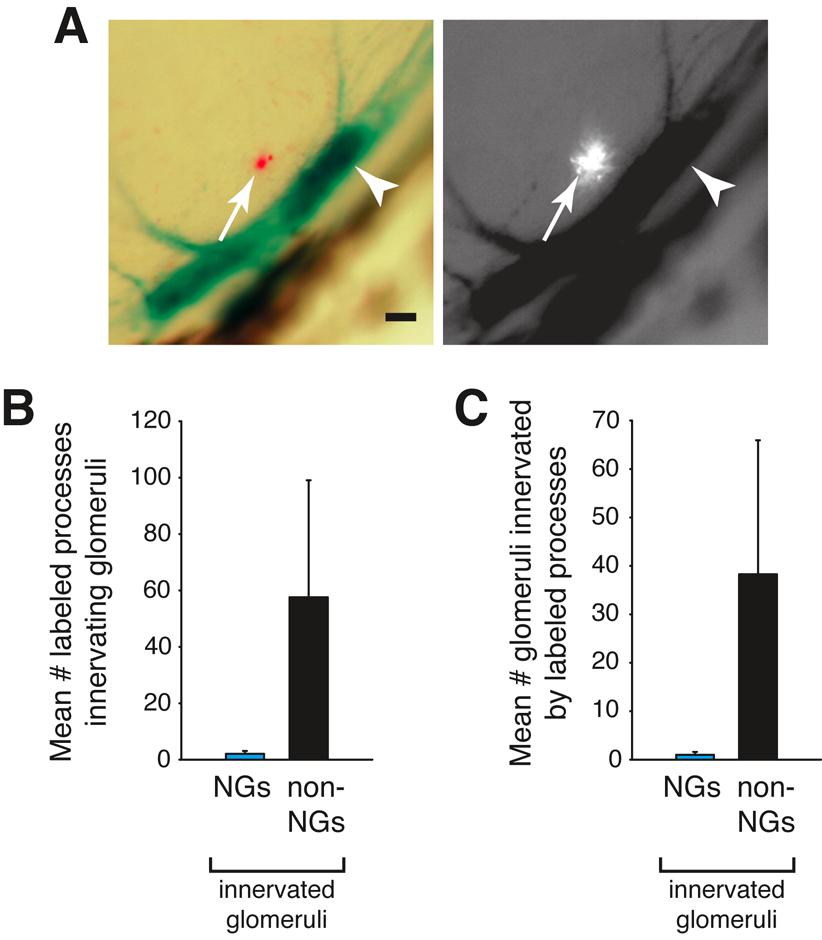

Supplement: Figure S3 — Cell labeling through DiI injections of non-necklace glomeruli. (A) Brightfield (left) and fluorescent (right) whole mount images after X-gal histochemistry of a non-necklace glomerulus in the posterior MOB injected with DiI (arrow). Arrowhead, nearby necklace glomerulus. Scale bar, 25 um. (B) Labeled cell processes innervating glomeruli (NGs, blue; non-NGs, black) after DiI injections of single beta-galactosidase-negative glomeruli (n = 4). Of these processes, 3.4% innervated NGs (2.0±1.0 processes per injection). Error bars, s.e.m. (C) NGs and non-NGs innervated by labeled processes after DiI injections of single beta-galactosidase-negative glomeruli (n = 4). Of innervated glomeruli, 2.5% were NGs (1.0±0.6 per injection). Error bars, s.e.m. (2.35 MB DOC) [file pone.0004657.s003.doc]
